# Molecular Regulation of Striatal Development: A Review

**DOI:** 10.1155/2012/106529

**Published:** 2012-01-26

**Authors:** A. E. Evans, C. M. Kelly, S. V. Precious, A. E. Rosser

**Affiliations:** Brain Repair Group, Cardiff University, Museum Avenue, Cardiff CF10 3AX, UK

## Abstract

The central nervous system is composed of the brain and the spinal cord. The brain is a complex organ that processes and coordinates activities of the body in bilaterian, higher-order animals. The development of the brain mirrors its complex function as it requires intricate genetic signalling at specific times, and deviations from this can lead to brain malformations such as anencephaly. Research into how the CNS is specified and patterned has been studied extensively in chick, fish, frog, and mice, but findings from the latter will be emphasised here as higher-order mammals show most similarity to the human brain. Specifically, we will focus on the embryonic development of an important forebrain structure, the striatum (also known as the dorsal striatum or neostriatum). Over the past decade, research on striatal development in mice has led to an influx of new information about the genes involved, but the precise orchestration between the genes, signalling molecules, and transcription factors remains unanswered. We aim to summarise what is known to date about the tightly controlled network of interacting genes that control striatal development. This paper will discuss early telencephalon patterning and dorsal ventral patterning with specific reference to the genes involved in striatal development.

## 1. Striatum: An Overview

**Figure 1:**
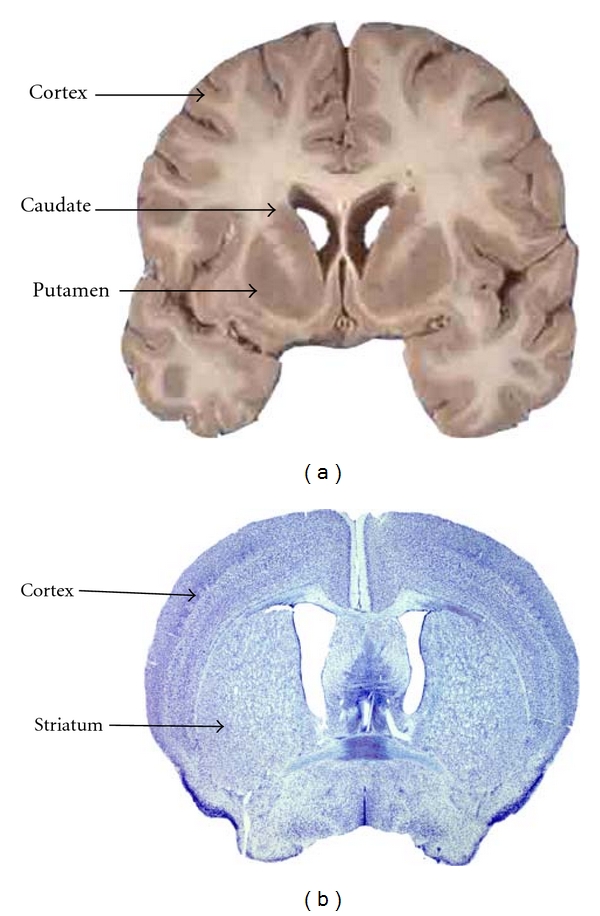
(a) Coronal section of a human brain showing the cortex, the caudate, and the putamen separately that when combined make up the striatum in comparison to (b) a caudal section of a mouse brain stained with cresyl violet showing the striatum as one structure and the cortex [1].

The striatum plays a vital role in the coordination of movement (primary motor control), emotions, and cognition [1–3]. In humans, the striatum is divided into two nuclei, the caudate and the putamen, by the internal capsule, whereas in mice it is one structure. This is shown in Figure 1. The complexity and importance of the striatum is best highlighted when it is impaired. There are a number of diseases that may produce striatal damage, including acquired conditions such as a stroke and genetically inherited conditions such as Huntington's disease (HD). HD is a condition that is characterised by neuronal dysfunction and neuronal loss that principally affects the medium spiny neurons (MSNs) of the striatum. MSNs are the major projection neuron and constitute the vast majority of neurons in this structure. HD results in progressive deterioration of movement and cognition and, in many cases, additional behavioural deficits over a period of 15–30 years, and eventually renders an individual unable to care for themselves. To date there is little in the way of symptomatic treatment and no disease-modifying agents available. A better understanding of striatal development is likely to accelerate our understanding of the pathogenic processes underlying conditions such as HD and is central to the development of protocols to engineer stem cells to be suitable as donor tissue for cell replacement therapy [3–5].

## 2. Neuronal Development

**Figure 2:**
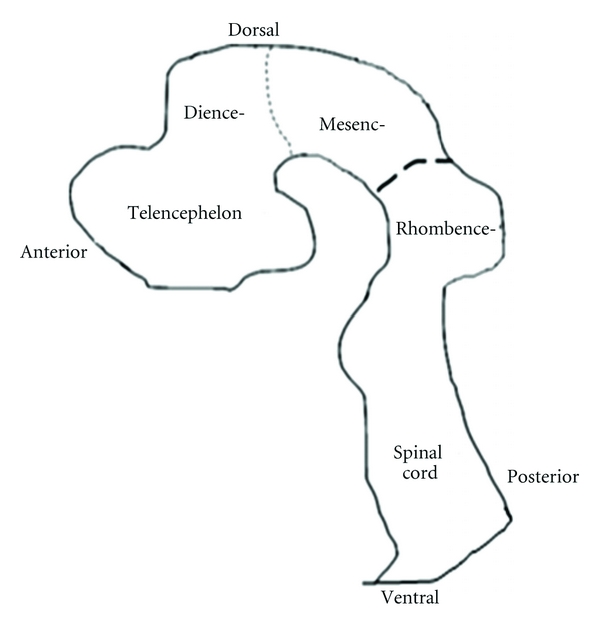
Patterning of the neural tube. The neural plate folds to form the neural tube, which comprises developing areas of the CNS. The prosencephalon is split into the telencephalon and diencephalon and the mesencephalon and rhombencephalon.

Development of the nervous system starts with neural induction, followed by neurulation that gives rise to the neural tube, and finally, patterning of this tube along the anterior-posterior (AP) axis. Following AP patterning, the neural tube folds and is subdivided into the prosencephalon (forebrain), the most anterior (rostral) part of the neural tube, which consists of the telencephalon and diencephalon, the mesencephalon (midbrain), and the rhombencephalon (hindbrain) [6]. These major subdivisions are shown in Figure 2. Regional patterning of the putative brain regions is then controlled by a series of interacting gene networks, of which the ones controlling telencephalic development are the most complex.

## 3. Regional Patterning of the Developing Telencephalon

The embryonic telencephalon, which is located at the most rostral end of the neural tube, is divided into the dorsal telencephalon (also called pallium), which gives rise to the neocortex, and the ventral telencephalon (also called the subpallium), which forms the striatum and is the origin of cells that populate the olfactory bulb, globus pallidus (GP), and some cells that also populate the cortex [7]. This paper will concentrate on the development of the ventral telencephalon. Although the adult striatum is different between all mammalian species, the initial subdivisions observed in the telencephalon are comparable [8, 9].

**Figure 3:**
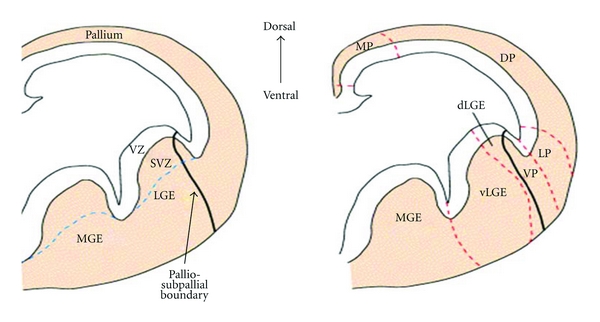
Coronal hemisections of the mouse telencephalon at E12.5 showing morphologically defined structures and the progenitor subdomains. The ventricular zone (VZ) extends along the DV axis and contains proliferative neuronal precursor cells. The subventricular zone (SVZ) (shown by the blue dashed lines) also contains precursor cells. Progenitor cells migrate radially and tangentially from these zones to populate the specific areas of the brain. The dashed red lines indicate the approximate boundaries between distinct telencephalon progenitor domains. Abbreviations: MGE/LGE medial/lateral ganglionic eminence; MP: medial pallium, DP: dorsal pallium LP: lateral pallium, VP: ventral pallium. Picture taken from [15].

Due to the rapid migration of postmitotic neurons in the subpallium, three prominent intraventricular bulges form; the septum, the medial, and lateral ganglionic eminences (MGE/LGE), collectively referred to as the whole ganglionic eminence (WGE), shown in Figure 3. The MGE, the most ventral eminence, gives rise to the amygdaloid body and the GP whilst the LGE, that is situated more dorsally, gives rise to the caudate and putamen [10, 11]. The LGE is further divided into the dorsal LGE (dLGE) and the ventral LGE (vLGE) on the basis of regional gene expression, which is discussed later.

Within the surrounding neural epithelium of the developing telencephalon, there are two proliferative zones, which are shown in Figure 3, the ventricular zone (VZ), which is positioned on the perimeter of the lateral ventricles, and the subventricular zone (SVZ) (unique to the telencephalon), which extends from the basal region of the VZ [12]. It is in the proliferative zones of the ventral telencephelon that both projection neurons (including MSNs) and *γ*-aminobutyric acid (GABAergic) interneurons are born before migrating to the regions they populate in the adult brain. Specifically, striatal projection neurons are born in the LGE and make up nearly 90% of LGE neurons. Interneurons originating from the LGE populate the cortex, olfactory bulb, and striatum, whereas those from the MGE migrate to the cortex, GP, and also the striatum [13–16].

## 4. Unzipping Telencephalon Development

The telencephalon is the most complex region of the mammalian brain and shows vast heterogeneity in terms of its various neuronal populations, structures, and function. Telencephalon development requires a variety of signals secreted from surrounding signalling centres to ensure the correct positional identity of the neurons, which will populate the adult forebrain. Several gene families are involved in coordinating the initial events for telencephalon patterning: principally fibroblast growth factors (*FGFs*), bone morphogenic proteins (*Bmps*), *Wnts *(originated from the drosophila gene *wingless*), retinoic acid (RA), and sonic hedgehog (*Shh*). These genes are responsible for activating downstream factors that enable signalling cascades to be initiated that allow cells to gain a positional and molecular identity [17]. It is likely that only a proportion of the factors required for neuronal identity have so far been identified, and the precise way in which such factors interact to specify the timing and terminal differentiation of particular neuronal subpopulations is not yet defined. However, there have been clear advances in knowledge in this area over the last few decades and we summarise here what is known about some of the key factors so far identified as being involved in striatal development.

## 5. FGF8

FGFs are ligands that activate several pathways, for example, Ras Map kinase (MAPK) pathway upon binding FGF receptors (FGFR) 1, 2, or 3 [18] and at least 5 FGF proteins have been indentified in CNS development [19]. *Fgf8 *is expressed rostrally from the anterior neural ridge (ANR) in mammals and has roles in proliferation and cell survival. In addition, it has been shown that *Fgf8 *regulates the expression of forkhead box protein G1 (FOXG1) (previously *bf1*), a rostral forebrain marker. It was shown that FGF8 could renew *Foxg1 *expression in mouse explants that had the ANR removed and secondly that inhibitors of *Fgf8 *reduced *Foxg1* expression in neural plate explants [20, 21]. In addition, reduction in *Fgf8* leads to rostral truncations and midline defects in the developing forebrain [22]. In *Fgf8 *null mice (*Fgf8^−/−^*), the telencephalon was smaller than in wild-type (WT) littermates and exhibited patterning abnormalities [22–24]. Specifically, loss of function studies showed that the MGE and LGE are absent, and there was loss of genes found in the ventral regions, for example, *Nkx2.1* and *Dlx2* and an expansion of the dorsal marker *Pax6 *[23]. These results suggest a role for *Fgf8* in ventralisation of the telencephalon. However, it seems unlikely that *Fgf8* is the sole factor involved in the induction of rostral forebrain development due to the continued presence of the telencephalon in *Fgf8^−/−^*or FGFR null mutants [22]. One explanation for the telencephalon still remaining in these mutants is that compensation is achieved by other *Fgfs *expressed at the same time. However, others in the field are of the opinion that overlapping *Fgf *expression profiles do not exist and that each *Fgf* has exclusive roles in telencephalon development [25, 26]. Therefore, the reason the telencephalon is not lost completely in the *Fgf8 *mutant is that *Fgf8* alone is not essential for telencephelon generation [26, 27]. However, the fact that beads soaked in FGF8 added to anterior neural explants lacking an ANR promote expression of *Foxg1* suggests FGFs are necessary for telencephalon induction [20].

Recently, work supporting the gain of function studies has allowed greater insight into the role of FGFs and FGFR in telencephalon development. Triple FGFR knockouts have shown that prior to telencephalon development at E10.5, embryos showed abnormalities in the anterior structures and by E12.5, a time when the telencephalic structures should be morphologically distinguishable, mutants lacked all anterior head structures and had no visible telencephalon except for the dorsal midline [19]. In addition, *Foxg1* was not expressed, together with complete absence of the ventral markers *Dlx2 *and* Nkx2.1*. Unexpectedly, the dorsal marker *Emx1* was also absent suggesting that FGF has a role in forming the dorsal telencephalon in addition to the ventral telencephalon [19].

The phenotype of the FGFR triple knockout was markedly more severe than the mild phenotype observed in single or double receptor mutants [18]. This supports the argument that these receptors do compensate for each other and do not have exclusive roles. However, this compensation is not absolute given that a mild phenotype was still evident in single or double mutants. This suggests the compensating receptors can function, but as they are not the first choice for the ligand, they produce signalling cascades at lower efficiencies [19]. Therefore, results from the different combinations of FGFR mutants suggest that the FGFR1 receptor is responsible for the majority of signalling, but it is the overall levels of FGF signalling that operate to initiate, pattern, and sustain early telencephalon development as a whole rather than specific ligands patterning different areas [19]. What is left to be answered is if the FGFs function as morphogens or independent to a concentration dependant gradient.

*Foxg1* and *Fgf8 *are both expressed at approximately the same time in the ANR (~E8) and function through a tightly linked positive feedback loop [19, 20]. FGF signalling is sufficient and necessary for *Foxg1* expression, and *Foxg1* is necessary for *Fgf8* expression [20]. However, the different phenotypes between *Foxg1* mutants and the FGFR triple mutant suggest that both genes must function at least partially through independent genetic pathways, the details of which are still unknown [19].

## 6. SHH

SHH is a member of the hedgehog (Hh) family of secreted proteins and acts as a morphogen that is first secreted from the notochord, which underlies the posterior structures of the brain, following which expression is from the overlying neural plate [6, 27, 28]. By E9.5 *Shh* is expressed in neural epithelium of the ventral telencephelon [29], and by E12 it is expressed in the mantle zone and is no longer detectable in the neuroepithelium [30]. *Shh *operates through a concentration gradient that spans the DV axis at different time points to confer different neuronal identities on the developing precursors, and expression is first seen in the ventral telencenphelon from E11.5 [31, 32]. SHH expression directs neural progenitors to a ventral fate and importantly is both necessary and sufficient to induce specific ventral forebrain markers [32–34]. *Shh* is expressed in the ventral telencephalon and is thought to maintain *Fgf8 *expression [32, 35] as well as induce expression of forebrain markers. Specifically, SHH activates several TFs including *Nkx2.1* [36, 37], *Gsx2* (formerly* Gsh2*) [38–40], and *Pax6 *[41].

**Figure 4:**
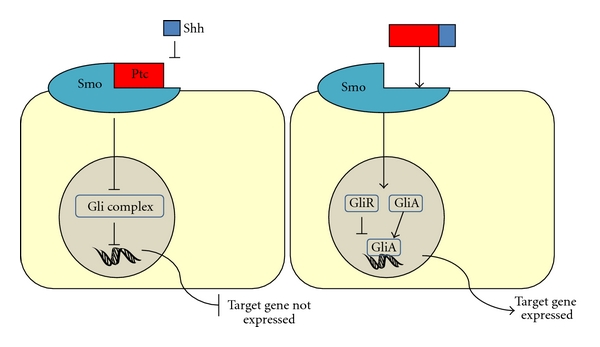
The Shh pathway for target gene expression: (a) repressed pathway—when SHH cannot bind Ptc, ptc represses gene expression by being bound to Smo. Smo cannot then activate the Gli complex meaning the target gene is repressed. (b) Induction pathway—when SHH binds ptc, smo is released which allows the GliA to bind the DNA and activate gene expression. Abbreviations: SHH: sonic hedgehog, Ptc: patched, Smo: smoothened, GliA: Gli Activator.

SHH acts as a ligand for a pathway involving two transmembrane proteins, patched (Ptc) and smoothened (Smo). Normally, Ptc is bound to Smo and the pathway is inactive as Smo is not free to activate the Glioma-associated oncogene homolog 3 (Gli3). This is illustrated in Figure 4. However, when SHH binds Ptc, Smo is de-repressed which results in the Gli repressor (GliR) becoming activated (GliA) and being able to translocate to the nucleus and activate gene expression, as shown in Figure 4.

There are three members of the Gli family of zinc- finger TFs, *Gli1*, *Gli2*, and *Gli3* and all have been shown to regulate SHH-dependant gene expression. Gli proteins have both activator and repressor activities, the N-terminal encodes a repressor function, and the C-terminal region is required for positive activity [43]. It is believed that the Gli3 protein functions principally in its repressor form and it appears that its activity is negatively regulated by *Shh *[44, 45], whereas Gli1 and 2 function primarily as transcriptional activators [46, 47]. The negative regulation by *Shh *on *Gli3* is observed in the limb, where *Shh* inhibits *Gli3* from processing into its repressor form [43]. Analysis of mouse mutants for each of the Gli genes (*Gli1^−/−^*), *Gli2^−/−^*, and *Gli3^−/−^*) has shown that mice lacking *Gli3* or *Gli2 s*how only slight defects in telencephelon development [48], whereas mice lacking Gli3 have strong defects in dorsal telencephelon patterning [49–51]. At the dorsal region of the telencephelon, where the concentration of SHH is limited, the *Gli3* protein is cleaved from an activator into a repressor form and promotes dorsal patterning [43]. It is the inhibition of the Gli3 repressor complex in the ventral region that facilities telencephalon development; therefore, the primary function of Shh is to prevent the production of excessive Gli repressors. However, as Gli3 has been shown to be able to function as a weak activator of Shh *in vivo* [52], the question recently investigated by Yu and colleagues [53] is whether this protein (or either Gli1 or 2) has an activating role in ventral telencephalon [53]. Recent work by this group suggests Gli activators do have a role in specification, differentiation, and positioning of some subgroups of telencephalon neuronal progenitors that arise from the interganglionic sulcus [53], but further experiments need to be carried out to learn more about the role of Gli proteins as activators, and therefore this paper will concentrate on the role of Gli3 as a repressor in DV patterning.

The relationship between *Shh and Gli3 *has been shown functionally through varying combinations of mutants. In *Shh* ^−/−^ mutants, the ventral markers *Dlx2*, and *Gsx2 *were reduced, whereas in *Gli3^−/−^* mutants the expression pattern of these genes was extended into more dorsal regions [43]. Overall the *Shh* ^−/−^ mutant shows more severe telencephelon abnormalities than the *Gli3^−/−^* mutant [49, 50]. In accord with the loss of ventral markers in Shh mutants (Shh−/−), there is a loss of ventral telencephalic cells leading to an altered morphology of the ventral telencephalon together with the ectopic expression of dorsal forebrain markers [33–35, 43]. Specifically, the mutants lack any MGE development as shown by the absence of Nkx2.1 [43]. *In vitro*, cultures of telencephalon explants treated with SHH result in expression of ventral markers such as Nkx2.1 [33]. The complimentary gain of function experiments carried out in both fish and mice has shown that SHH promotes ventral identity in dorsal telencephalic cells *in vivo* with induction of the ventral forebrain markers Gsx2, Dlx2 and Nkx2.1 [38, 54]. Importantly, conditional knockouts using a FoxG1-Cre to knock-out Shh have allowed the optimal window of signalling in telencephalon development to be investigated. Fuccillo et al. [55] showed that if Shh is knocked-out at E8.5, there are severe defects of all ventral telencephalic regions [55]. However, in knockouts at E10-12 using a Nestin-Cre, there are limited defects in ventral telencephalic patterning and cortical interneurons are affected rather than gross patterning deficits [56].

In the Shh−/− and Gli3−/+ mutants, telencephalon morphology is largely restored to WT, but regional gene expression is not fully restored. The ventral marker Nkx2.1 is not rescued unless both copies of the Gli3 protein are removed, suggesting Nkx2.1 is very sensitive to the antagonism between Shh and Gli3 [43]. Also, in Gli3−/− mutants, the expression of Nkx2.1 is not extended dorsally like Dlx2 and Gsx2, nor can it be expressed ectopically in the cortex when exogenous Shh is added. From this Rallu and colleagues suggested that the Gli2 protein has a complimentary role to Gli3. It is thought that Gli2 functions as a weaker repressor and can repress the expansion of Nkx2.1 in Gli3−/− mutants but is not strong enough to prevent the expansion of the more ventral genes Dlx2 and Gsx2 [43]. Moreover, in the Shh−/− and Gli3−/−, double mutant ventral markers such as Gsx2 and Dlx again were largely restored to WT levels [43, 57]. In support of this, Nkx2.1 expression was expanded in Ptc mutant mice (Ptc−/−). In this mutant, Smo should be persistently de-repressed and Gli proteins should remain in their activated form, thus having a repressive effect on Nkx2.1 expression in the MGE [58]. However, the fact that Dlx2 and Gsh2 were restored in the absence of Shh signalling in this mutant suggests that other genes and signalling pathways, independent to Shh signalling, have a role in DV patterning of the telencephalon [43].

In summary between E9 and E12.5, SHH acts mainly by inhibiting the formation of the Gli3 repressor [43] and contributes to the establishment of DV patterning [34, 55]. Secondly, SHH signalling also supports the expansion of progenitors of the ventral telencephalon by inducing and maintaining the expression of *Nkx2.1* until at least E14 and later into neurogenesis [56].

## 7. Retinoic Acid

RA is the biologically active form of vitamin A and has been implicated in survival, specification, proliferation, and differentiation during forebrain development [58–60]. Two oxidation events occur to ensure RA is successfully derived to function as a ligand for either RA receptors (RARs) (RAR*α*, RAR*β* and RAR*γ*) or retinoid X receptors (RXR*α*, RXR*β*, and RXR*γ*) that belong to the steroid/thyroid receptor superfamily [61]. Initially retinol dehydrogenases oxidate retinol to retinaldehyde and then the rate-limiting enzymes, retinaldehyde dehydrogenases (Raldh), are required to oxidate retinaldehyde to RA [62].

The first known source of RA in the developing striatum is in the LGE at approximately E12.5 and is produced from reactions mainly catalysed by Raldh3 [63]. It is not until E14 that RA and Raldh3 are obviously expressed in the LGE and promote GABAergic neuronal differentiation by inducing Gad67, an enzyme needed for GABA synthesis [64]. Raldh3 continues to promote GABAergic differentiation at E18.5, and RA continues to be expressed into adulthood [64]. It has also been reported that *in vitro* LGE-derived neurospheres and human embryonic stem cells (hESCs) induce GABAergic differentiation once RA was added to the media [64]. However, it is thought that the RA in the LGE is not only a product from Raldh3-mediated reactions as Raldh3^−/−^ mutant mice do not show an obvious telencephalic phenotype [65]. It has been shown that retinoids from the glia found in the LGE are also functioning in striatal neuronal differentiation [66]; therefore, it is possible that these, and other sources not yet known could be compensating for Raldh3.

 Furthermore, at this time the RARs *α* and *β* are present in the ventral telencephalon, with RAR*β* preferentially expressed in LGE together with RXR*γ* [67]. In RAR *β* ^−/−^ mutant mice, there is a loss of striatal-enriched tyrosine phosphatase mRNA, a gene regulated by RA in the striatum [67], and a reduction of striatal dopamine and cAMP-regulated protein (DARPP-32) positive neurons together with dynophin, *μ*-opioid receptor (MOR1), and tyrosine hydroxylase (TH) compared to WT mice [59, 67]. It has also been shown that when chick explants of LGE are treated with RAR antagonists, LGE specification is prevented [58]. The complimentary experiment shows that when exogenous RA was added to dorsal explants, LGE was evident instead of MGE [58]. Additionally, supplementation of RA to LGE cultures showed an increase in DARPP-32 positive neurons, whilst there was no effect seen in the MGE relevant to increasing doses of RA [66]. Moreover, blocking RA in chick embryos prevents the expression of *Meis2* which is expressed in progenitor cells of the intermediate zone of the telencephalon and is the earliest known marker of striatal precursors [66]. Taken together, these results confirm the importance of RA in LGE specification.

As well as being important in embryonic development, RA expression remains in the forebrain throughout adult life and has been shown to maintain the expression of *Fgf8* and *Shh* in this region, as when RA is removed, *Fgf8* and *Shh* expression is lost [59, 60]. It has recently been proposed that *Nolz1*, a zinc finger TF that is expressed in the proliferative SVZ in LGE precursor cells, is implicated in RA signalling [68]. At E12.5 *Nolz1*-induced neurogenesis partially depends on RA signalling as it has been shown that this TF activates the RAR *β* receptor in LGE-derived neural precursor cells and that this effect was inhibited when RA was removed [68]. However, *Nolz1* expression was not affected in Raldh3 mutant mice (Raldh3^−/−^), which lacks RA in the LGE, or when a vitamin A deficient diet was fed to the mothers [63, 69] suggesting RA is not essential to *Nolz1* expression throughout development and is only needed to induce early expression. RA activates *Nolz1* to induce initial neurogenesis during early striatal development at E12.5 but is not sufficient for its maintenance beyond this time [68]. Also, it has also been shown that *Nolz1* contributes to later striatal development by working downstream of *Gsx2* to activate the RAR*β* receptor.

## 8. Wnt Signalling

**Figure 5:**
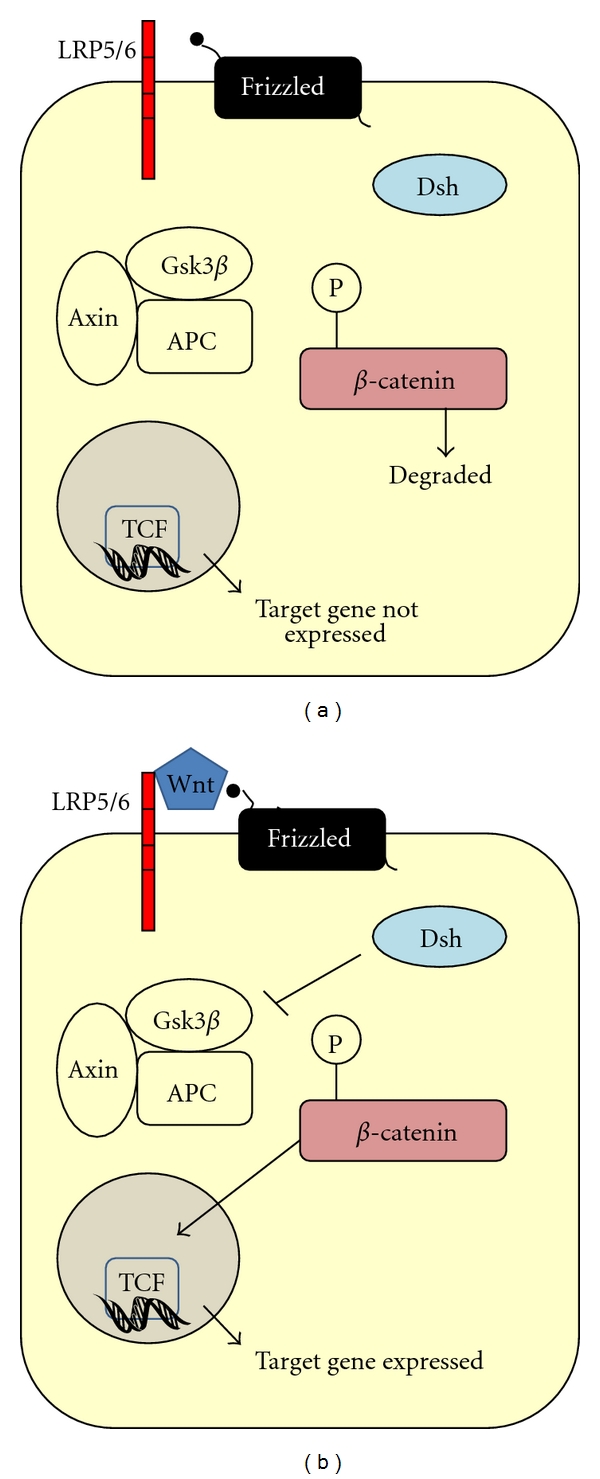
The canonical Wnt pathway for target gene expression. (a) When a WNT ligand is absent, *β*-catenin is phosphorylated by the Gsk3*β* complex and is targeted for degradation and there is no gene expression. (b) When a Wnt ligand binds Frizzled, Dsh is activated and inhibits the axin-Gsk3*β* complex phosphorylating *β*-catenin enabling it to translocate to the nucleus and can activate TCF which can activate gene expression. Abbreviations: APC: adenomatous polyposis coli, Dsh: Dishevelled; GSK3*β*: glycogen synthase kinase 3 *β*; TCF: T-cell factor.

Wnts belong to the wingless protein family and are a class of ligands that are crucial in embryogenesis and have been implicated in CNS development. Wnts can signal through three different pathways: the canonical pathway, the planar cell polarity pathway, and the calcium pathway and it is the canonical pathway that is important in telencephalon development. In the canonical pathway, *β*-catenin is indirectly activated by a WNT ligand binding to the cell surface receptor, Frizzled. Upon binding, frizzled activates its intracellular component dishevelled (Dsh) that dephosphorylates *β*-catenin preventing its degradation by the axin-glycogen synthase kinas 3*β* (GSK3*β*) complex (in the absence of WNT signalling, *β*-catenin is phosphorylated by the GSK3*β* complex and degraded). *β*-catenin then translocates to the nucleus where it can activate the transcription of *Wnt* target genes such as T-cell factors (TCF), which in turn regulate genes such as c-myc. This pathway is shown in Figure 5.

Wnts are part of the cohort of caudalizing factors that are involved in the initial AP orientation of the neural plate and are crucial for the generation of the dorsal telencephelon [70]. Specific concentrations of WNTs are needed to further refine regional patterning and to induce the expression of *Pax6,* a dorsal telencephelon marker [71]. A reporter line carrying a *LacZ* reporter gene under the control of *β*-catenin/TCF response elements showed that WNT signalling is active in the pallium at E11.5 and E16.5 but not in the subpallium [72, 73]. In the absence of canonical signalling, there was ectopic expression of *Gsx2, Dlx2*, and *Ascl1 *(formerly *Mash1*) in dorsal telencephelon together with downregulation of the dorsal markers *Emx1, 2* and *3 *[73]. This ectopic expression of ventral genes facilitated the cells of the dorsal telencephelon to adopt a ventral fate, therefore allowing these cells to have the potential to become GABAergic projection neurons [73]. Therefore, this work in mice has shown that WNT signalling is necessary for ensuring the correct molecular characterisation, and thus morphology, of the dorsal telencephelon before the onset of neurogenesis and that inhibition of WNT signalling is necessary for subpallidal development [73]. In addition, gain of function experiments carried out in chicks has shown the importance of *Wnt *gene expression in dorsal telencephelon patterning. Using chick explant cultures, Gunhaga et al. [71] showed that *Wnt3a* or *Wnt8* expression can convert the ventral telencephalic cells into *Pax6 *and *Ngn2* positive cells at the expense of *Ascl1 *and *Nkx2.1 *[71].

## 9. BMPs

Although BMP inhibition is required for neuronal development, graded concentrations are necessary in the neural plate to establish medial-to-lateral patterning [42]. BMPs belong to the TGF*β* family of secreted proteins. It is thought that BMPs are also needed to dorsalize the telencephalon and restrict ventral telencephalic development. Forebrain patterning was repressed in forebrain explant cultures when BMPs were added as shown by inhibition of *Foxg1, Nkx2.1*, and *Dlx2 *[74]. Similarly beads soaked in BMP4 or BMP5 that were implanted into the neural tube of a chick forebrain induced dorsal markers, for example, Wnt4 and repressed ventral markers [75]. Additionally, when the telencephalic roof plate (a source of BMPs) was ablated, there was a reduction in cortical size and a decrease of one of the most dorsal cortical markers, *Lhx2 *[76]. BMPs are inhibited by several factors including chordin and noggin. In mice that lacked both copies of the chordin gene (*chordin* ^−/−^) and one copy of the noggin gene (*noggin* ^+/−^), a dorsal, rather than ventral telencephalon was evident. However, this effect may not be direct because of an increase in BMP and may be in part due to the decreased levels of *Shh* and *Fgf8 *expression in the forebrain caused by increased BMP levels [77].

## 10. Foxg1

*Foxg1* is a member of the winged helix family of TFs and is the earliest recognised marker of the telencephalon [78]. This TF was first identified in the rat brain, where it was shown that its expression was restricted to the telencephelon [78]. By E8.5 *Foxg1* is expressed in the neural tube, specifically in the anterior plate cells that are fated to contribute to the telencephalon [20, 79], and functions to establish and subdivide the telencephalon.

*Foxg1^−/−^* mutant mice show no morphological differences in the size of the developing telencephalon at E10.5. However, the ventral markers *Ascl1, Nkx2.1, Gsx2*, and *Dlx1/2* are absent, and instead the dorsal markers, *Emx2* and *Pax6*, are expressed throughout the telencephelon [80, 81]. It has also been shown that *Fgf8* was reduced in the mutant telencephalon at E10.5 [81]. By E12.5 there were considerable morphological differences in the ventral telencephalon of the mutant when compared to WT; notably the GEs were absent but there were no defects in the dorsal area [80, 81]. A reason for the absence of the ventral telencephalon in these mutants is the loss of proliferating cells in this region (shown through BrdU staining) with telencephalic proliferation being restricted to the dorsal region [80]. This decrease in proliferation could be a direct consequence of the lack of *Foxg1 *expression or could be due to effectors of *Foxg1* not being able to function optimally.

It has also been shown that *Foxg1 *coordinates signalling pathways of SHH and WNTs, which are required for the development of the subpallial and pallial telencephalon, respectively, and have been described above [82]. Dorsal identity is prevented by *Foxg1 *inhibiting the Wnt pathway, further confirming the role of *Foxg1* in ventral telencephalon induction in an independent way to SHH. Manuel et al. [17] cultured cells from* Foxg1^−/−^* mice and showed that the addition of SHH and FGF8 alone could not induce the expression of other ventral telencephalon genes. Also, further experiments where *Foxg1^−/−^* cells were grafted into *Foxg1^−/−^* /* Foxg1^+/+^*chimeras, showed that the mutant cells, were specified abnormally, did not integrate with WT cells and expressed dorsal rather than ventral markers [17]. Recently, Manuel et al. [83] have also shown that the reason for mutant cells behaving differently is due to them having an increased cell cycle, something initially reported by Martynoga et al. [81]. Specifically, Manuel and colleagues have shown that this is due to a decrease in *Pax6 *expression, a cell cycle organiser. Upon addition of PAX6, the mutant phenotype was partially rescued [83]. This work suggests that *Foxg1* not only promotes the production of ventralising cues, but has a cell autonomous role in regulating *Pax6* [83]. *Foxg1* is crucial in forebrain development and is absolutely required for the regulation of telencephalic identity.

## 11. Dorsoventral (DV) Organisation of the Developing Telencephalon

**Figure 6:**
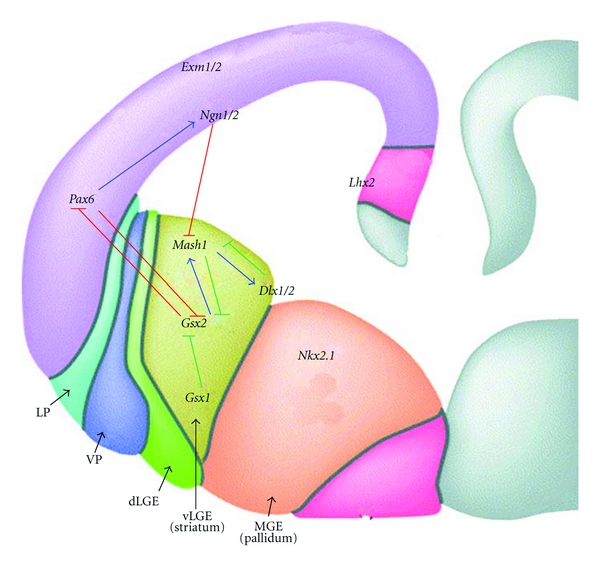
Schematic coronal section through the developing telencephalon at E12.5—the dorsal and ventral subdomains are shown and defined by unique gene expression patterns. Dorsal telencephalic markers shown are *Emx1/2, Ngn1/1*, and *Pax6*. The ventral telencephalic markers shown can be split into identifying the LGE or MGE. *Mash1(Ascl1), Gsx1/2*, and *Dlx1/2* are associated with the LGE (specifically the vLGE), and *Nkx2.1* is associated with the MGE. Some of the gene interactions are shown on the diagram. One of the important interactions is between *Pax6* and *Gsx2*; these genes work together to ensure the subpallalial/pallial border is maintained. Arrows denote positive interactions; T-bars denote inhibitory control. The green arrows represent the genetic signalling that has been unravelled in more recent data and occurs at later time points. Figure adapted from [42].

As discussed already, the developing telencephalon is divided into the dorsal region, and the ventral region and these areas can be defined on a morphological and genetic basis. In the dorsal telencephalon *Pax6*, *Neurogenin (Ngn) 1/2* and *Emx1/2* are expressed; in the ventral telencephalon, *Gsx2, Asc1, Dlx1/2*, and *Nkx2.1* are expressed, as shown in Figure 6 [84]. The pallial genes will only be discussed briefly in the context of them as markers, not to fully elucidate their role in cortical development. *Emx1/2* expression profiles are restricted to the most dorsal region of the cortex with no expression seen in the ventral cortical region. *Ngn 1* and *2* are basic helix loop helix (bHLH) TFs which are expressed throughout the cortex together with *Pax6*. In the absence of *Ngn* expression, *Ascl1* is ectopically expressed in the dorsal telencephalon thus priming these cells to adopt a ventral fate and becoming GABAergic rather than glutamatergic neurons. Therefore, the role of *Ngn1/2* is to maintain the DV boundary in the developing telencephalon and to inhibit ventral gene expression such as *Ascl1 *[24, 85].

Homeodomain gene interactions are crucial in mediating DV patterning and importantly, in setting up regional subdivisions within the developing telencephalon, as well as within the ventral telencephelon, and are principally regulated through *Shh* [36–41]. *Pax6* and *Gsx2 *are two members of this homeodomain family [86]. These proteins have overlapping expression profiles and work in synergy to ensure the subpallium-pallium border is maintained [39]. Their expression profiles mirror each other; Pax6 is expressed in a dorsal (high) to ventral (low) gradient and *Gsx2* is expressed in a ventral (high) to dorsal (low) gradient [87].

The embryonic patterning role of *Pax6 *was initially identified through genetic mapping of the classical “small eye” (*sey*) mouse mutant [88]. *Pax6* is needed for cortical development and to establish the subpallial-pallial border and is initially detected in the developing forebrain at E8 [89]. It is not until the neural tube stage that expression is downregulated in ventral regions simultaneous with the up regulation of *Nkx2.1* in this region, thus instantaneously setting up the DV ventral border on the basis of differential gene expression [37, 38, 90]. In *Pax6* mutant mice (*Pax6^−/−^*), there is a shift in the cortical-striatal boundary [91] and the cortical markers *Ngn1/2* and *Emx1* are downregulated, and *Dlx1/2 *[39, 41, 92],* Ascl11* [39, 41] and *Gsx2* [39] are ectopically expressed in dorsal regions of the telencephalon. *Nkx2.1* also expands dorsally into the LGE shifting the LGE-MGE border [41].

*Gsx2 *is first detected in the developing forebrain between E9 and E10 and is expressed in the LGE. *Gsx2* mutants (*Gsx2^−/−^*) have the opposite phenotype to that seen in *Pax6* mutants; there is ectopic expression of *Pax6* and *Ngn2* in the LGE accompanied by the loss of *Ascl1* and *Dlx2 *[38–40]. On the whole, there was a reduction in the size of the LGE at E12 [93], which by E18.5 led to a reduction in the size of the striatum [94] and reduced expression of striatal projection neurons confirmed through a marked decrease in DARPP-32 and the earlier MSN marker, Forkhead box P1 (*FoxP1*) [38–40, 95]. However, there was a slight increase in the striatal-matrix marker calbindin [95]. These results suggest that *Gsx2* is a crucial inducer of *Ascl1, Dlx1*,* and Dlx2* whilst repressing dorsal character and is implicated in the differentiation of calbindin positive neurons. However, in mice that lack both *Gsx2* and *Pax6* (*Gsx2^−/−^* and *Pax6^−/−^*), the phenotype observed was more subtle than the single mutations as is the case in *Shh* ^−/−^ *Gli3^−/−^* double mutants [38]. This suggests that other genes are also important in mediating DV patterning and positioning of the pallial-subpallial boundary.

*Gsx1*, a gene closely related to *Gsx2*, is also expressed in the ventral telencephalon but unlike *Gsx2* its expression is restricted to the ventral most region of the LGE [96]. It is thought that *Gsx1 *can partially compensate for the phenotype observed in *Gsx2^−/−^* mutants [95, 97, 98]. In the *Gsx2^−/−^* mutant, *Gsx1* expression spreads throughout the LGE between E11 and E14.5 and shows similar expression patterns to *Ascl1* in the LGE [95]. DARPP-32 expression is lost in both *Gsx2^−/−^ /Ascl1^−^* ^/−^ and *Gsx2^−/−^ Gsx1^−/−^* double mutants suggesting *Ascl1* has a role in regulating *Gsx1* to compensate for *Gsx2* in Gsx*2^−/−^* mutants [95]. Until recently the role of *Gsx1* has remained elusive as no phenotype has been discovered through using knockouts. Pei et al. [99] have shown that *Gsx1* and *Gsx2* differentially regulate the maturation of LGE progenitors. Gain-of-function experiments were carried out to distinguish if these two closely linked genes do indeed carry out different roles in the LGE or if *Gsx1* is simply a “backup” for *Gsx2.* Results showed that *Gsx2* maintains LGE progenitors in an undifferentiated position before *Gsx1*, in part through the downregulation of* Gsx2, *directs the progenitors to acquire a mature neuronal phenotype [99]. This is shown in Figure 6. These novel results indicate that the *Gsx* genes regulate LGE patterning through a controlled balance of signalling allowing proliferation and differentiation of neuronal progenitors [99].

*Ascl1* is also a member of the bHLH family of TFs. It has a primary role in the correct development of the ventral telencephalon and relies on *Gsx2* for normal expression [38–40, 100]. In contrast to *Ngn1/2* that is expressed in the dorsal telencephelon, *Ascl1* is expressed throughout the ventral telencephalon and ASCL1 protein is seen in the VZ and SVZ, where neuronal precursor cells reside [101]. When *Ascl1* was ectopically expressed in the dorsal telencephalon, it was able to induce neurons to express Dlx1/2 at the expense of cortical markers [24]. *Ascl1* interacts with *Dlx1/2* that in turn activates GAD/67, the rate-limiting enzyme for GABAergic synthesis, and the two combined function to facilitate GABAergic differentiation in the telencephalon [85, 100]. However, in *Ascl1* knockout experiments (*Ascl1^−/−^*), *Dlx* and *Gad/67* are still expressed in the ventral telencephelon [100]. Together with the fact that these developing neurons can still acquire a GABAergic phenotype in the absence of *Dlx1* and *2*, this suggests an element of redundancy in this signalling pathway and/or the involvement of other genes not yet identified. Expression of *Gsx2* in the Ascl1^−/−^ mutant is the same at E12.5, but by E18.5 there is an increase in *Gsx2* expressing cells suggesting that *Ascl1* has the additional role of repressing *Gsx2* function later in development. This is shown in Figure 6 [94]. *Ascl1^−/−^* mutants also show a reduction in the number of early born striatal (cholinergic) and cortical (GABAergic) interneurons and a reduction in size of the MGE [100]. This phenotype can be explained by the initial loss of precursor cells in the SVZ, subsequently leads to a decrease in neurons populating the mantle zone [100, 102]. Conversely, TH, D2R, and enkephalin positive neurons are only slightly reduced in this mutant. This is expected considering the LGE is only partially reduced [102]. However, Wang et al. [94] suggest that the reason for the *Ascl1* mutant not giving a more severe phenotype is that *Gsx2* signals through another bHLH TF.

From these experiments, it can be concluded that *Ascl1* has the dual role of specifying precursors and controlling the timing of their differentiation, principally in the MGE and possibly has a role in the LGE, although it is not crucial in this later eminence [98, 102]. Recent experiments by Castro and colleagues have looked more closely into the precise mechanisms by which *Ascl1* controls proliferation of neuronal precursors [103]. Gene expression analysis carried out from embryonic brains and neural stem cell cultures showed that *Ascl1* has a role in regulating genes concerned with cell cycle progression and ultimately showed that there was a direct association between neural progenitor expansion and the corresponding phases of cell cycle exit and neuronal differentiation [103]. What is clear is that *Ascl1* is autonomously involved in patterning of early telencephalic progenitors (~E10.5) and nonautonomously involved in repressing the differentiation of adjacent progenitors through Notch signalling [100]. After *Ascl1 *has been expressed to aid neurogenesis*, Dlx1* and *2* repress *Ascl1* and subsequent notch signalling to promote terminal neuronal differentiation [104].

The Dlx family bears homology to the drosophila distal less-homeobox gene family of which there are 6 murine members, 4 of which are expressed in the developing MGE and LGE [105]. *Dlx1* and *Dlx2 *are expressed by subsets of cells in the VZ and by most cells in the SVZ and switch off as cells start to differentiate [101, 106]. *Dlx5 *and *Dlx6 *are expressed in the SVZ and mantle zone only [13]. Single mutations of *Dlx1* or *2 *show no noticeable forebrain defects; although in the absence of both *Dlx1 *and* 2*; there is arrested migration of matrix neurons within the SVZ [13, 106]. However, striatal development is not stopped completely, and this phenotype suggests other genes are involved in neuronal migration or that other genes such as *Gsx2* and *Ascl1* are compensating somehow by directly activating the targets of *Dlx1 *and *2*. A series of experiments from Rubenstein's lab have sought to identify other genes and TFs that could function downstream and upstream of *Dlx1/2* to control LGE specification and differentiation through a series of gene expression arrays and *in situ *hybridisations [107]. It is likely that further work will continue to find relationships between these genes and Dlx*1* and *2.*

As mentioned above, *Dlx1* and *2* activate GAD67, an enzyme needed for GABA synthesis and found in neuronal precursors of the SVZ and neurons of the mantle zone in the ventral telencephalon [100], and in *Dlx1/2^−^* ^/−^ there are decreased levels of GAD67 in the dLGE [108]. It is has been suggested that *Dlx1/2* indirectly activate GAD67 and that cooperation with other proteins is needed to promote a GABA neuronal phenotype [109]. Necidin is a maternally imprinted gene and is only expressed in the paternal allele [110, 111]. Mutant mice that lacked the paternal necidin allele showed a significant decrease in the differentiation of GABAergic neurons *in vivo* and *in vitro*, therefore suggesting that necidin facilitates the specification of GABAergic neurons in cooperation with Dlx proteins [109].

*Nkx2.1* is expressed exclusively in the MGE and is another homedomain protein. The primary role of Nkx2.1 is in ventral specification of the telencephalon where it acts to repress LGE identity, and it is also important in the development of striatal interneurons [7, 37]. *Nkx2.1* is induced by *Shh *at E8 [33], and as earlier mentioned, inhibition of *Shh *leads to reduced expression of *Nkx2.1* and dorsalisation of the ventral embryo [34]. In the *Nkx2.1* mutant mouse (*Nkx2.1^−/−^*), there are a lack of MGE derivatives and a DV switch of the MGE as it shows properties similar to the LGE rather than the MGE, for example some cells have been shown to express DARPP-32 [37]. The loss of *Nkx2.1* also showed a reduction of GABA and calbindin positive neurons from the cortex [37]. Additionally, generation of an (*Nkx2.1^−/−^*) mouse with the addition of a DlxtauLacZ reporter gene showed loss of early migration of *Dlx2*-expressing progenitors [30].

It is clear that DV patterning of the telencephelon requires the precise orchestration of several genes and TFs that work together to ensure the correct development of the striatum. It is likely that further experiments using the ever expanding range of genetic tools will further disclose the roles of genes already known, further elucidate the signalling pathways they operate through, and identify novel genes that have a functional role in striatal development and that can be used as specific regional markers [112–114].

## 12. Conclusion

This paper has aimed to summarise and organise research that is being carried out to understand the genetic mechanisms controlling striatal development. However, it is clear that there are still many pieces of the jigsaw to be found and fitted into the gaps of this puzzle. The more that is known about the development of the striatum, and, importantly, the development and differentiation of striatal MSN neurons, the more precise the protocols can be to direct the fate of renewable cell sources, such as embryonic stem cells, to a functional MSN phenotype for use in cell replacement therapy for HD. An additional aspiration is to identify specific genes to detect MSN precursors rather than relying on markers of terminally differentiated MSNs, such as DARPP-32. Earlier markers of putative MSNs could be used to facilitate the generation and refinement of neuronal differentiation protocols as well as tracking neuronal differentiation in grafts.
